# A205 IS REPEAT ERCP REQUIRED AFTER INITIAL ENDOSCOPIC MANAGEMENT OF POST-SURGICAL BILE LEAKS? MULTI-CENTER VALIDATION OF THE CALGARY BILE LEAK RULE

**DOI:** 10.1093/jcag/gwab049.204

**Published:** 2022-02-21

**Authors:** Y Xiao, M Salim, Z Meng, U Khan, A R Kohansal, N Forbes, S Heitman, P D James

**Affiliations:** 1 University of Toronto, Toronto, ON, Canada; 2 University Health Network, Toronto, ON, Canada; 3 University of Alberta, Edmonton, AB, Canada; 4 Gastroenterology, University of Alberta, Edmonton, AB, Canada; 5 University of Calgary, Calgary, AB, Canada

## Abstract

**Background:**

The Calgary Bile Leak Rule was developed to identify patients in whom biliary stent removal via gastroscopy could be safely performed in lieu of ERCP for post-surgical bile leaks.

**Aims:**

This study aimed to evaluate a Modified Calgary Bile Leak Rule (MCBLR) for a cohort of patients who underwent laparoscopic cholecystectomy complicated by bile leak.

**Methods:**

This retrospective cohort study included patients who underwent ERCP for management of laparoscopic cholecystectomy-induced bile leaks between 2005 and 2017. The primary outcome was defined as the absence of persisting bile leak or other pathology on follow-up ERCP. The MCBLR includes a) normal post-surgical serum alkaline phosphatase, b) small or absent leak with no other biliary pathology on initial ERCP, and c) time between initial and follow-up ERCP was 4–8 weeks. Test performance of the prediction rule was analyzed by calculating sensitivity, specificity, positive predictive value and negative predictive value.

**Results:**

124 cases met inclusion criteria, of which 116 (94%) of bile leak cases had no leak identified during the follow-up ERCP. 8 (6.4%) had a persisting bile leak on follow-up ERCP. Bivariate analysis found no factors significantly associated with the primary outcome. The MCBLR demonstrated a sensitivity of 100% (95% CI 63% - 100%), a specificity of 35% (95% CI 26% - 44%), a positive predictive value of 10% (95% CI 4% - 18%), and a negative predictive value of 100.0% (91% to 100%).

**Conclusions:**

The MCBLR demonstrated high sensitivity and negative predictive value for determining the need for repeat ERCP following endoscopic management of laparoscopic cholecystectomy-induced bile leaks.

**Funding Agencies:**

None

